# Clinical and dermoscopic patterns of acquired melanocytic nevi in children and adolescents: a cross-sectional study from Turkey^☆^

**DOI:** 10.1016/j.abd.2024.04.005

**Published:** 2024-10-31

**Authors:** Zeynep Keskinkaya, Özge Kaya, Selda Işık Mermutlu, Hilay Garipcan Karaemir, Sevilay Oğuz Kılıç

**Affiliations:** a Department of Dermatology and Venereology, Çanakkale Onsekiz Mart University Faculty of Medicine, Çanakkale, Turkey; b Department of Dermatology and Venereology, Medipol Mega University Hospital, İstanbul, Turkey

**Keywords:** Adolescent, Child, Dermoscopy, Nevus

## Abstract

**Background:**

Childhood and adolescence are the most active periods for nevi development, which provide insights into nevogenesis.

**Objectives:**

To evaluate the clinical and dermoscopic characteristics of acquired melanocytic nevi in Turkish children (aged ≤ 10-years) and adolescents (aged > 10-years) regarding demographic, constitutional, and environmental factors.

**Methods:**

A cross-sectional study on participants aged < 18-years examined for acquired melanocytic nevi between January and June 2023.

**Results:**

One hundred participants (female: male ratio = 1:1; median age: 10) were assessed. The median nevi number was significantly higher in adolescents than in children (6 vs. 4; p < 0.05). The upper extremities (n = 68) and trunk (n = 67) were the most commonly involved anatomical regions. Females had a significantly higher nevi rate on the upper extremities than males (80% vs. 56%; p < 0.05). The trunk was involved slightly more frequently in males (76% vs. 58%; p = 0.06). The globular pattern rate was higher in children than in adolescents (70.6% vs. 42.9%; p < 0.05), whereas a striking increase was observed in the reticular pattern from childhood (2%) to adolescence (14.3%) (p < 0.05). The globular pattern was the major dermoscopic pattern in all anatomical locations except lower extremities where the homogeneous pattern prevailed. Sunscreen use had no impact on the nevi number or dermoscopic pattern.

**Study limitations:**

Limited number of participants.

**Conclusions:**

The age and anatomical site were the most relevant factors influencing the number and dermoscopic patterns of nevi. The gender-related distribution pattern of nevi, without any effect of sunscreen use on either nevus count or dermoscopic pattern, suggests a genetic predisposition.

## Introduction

The melanocytic nevi are both well-known risk factors and mimickers of melanoma.^1^, ^2^, ^3^ During the last three decades, the clinical features and anatomical distribution patterns of nevi in the pediatric age group and their association with demographic, constitutional, and environmental factors were widely investigated.^4^, ^5^, ^6^, ^7^, ^8^, ^9^, ^10^, ^11^, ^12^

Following the advent of dermoscopy into the routine clinical practice, increasing reports on dermoscopic features of nevi during childhood and adolescence, the most active periods in terms of nevus development,^13^ have created a basic knowledge.^3^, ^14^, ^15^, ^16^, ^17^, ^18^, ^19^, ^20^, ^21^, ^22^, ^23^, ^24^, ^25^ In order to understand the concept of nevogenesis, special effort has been invested in age-related dermoscopic patterns of melanocytic nevi.^3^, ^14^, ^15^, ^16^, ^17^, ^18^, ^19^, ^20^, ^21^, ^22^, ^23^, ^24^, ^25^

Being familiar with the benign dermoscopic patterns would guide proper clinical management of nevi, ease early diagnosis of melanoma, and avoid unnecessary biopsies performed to rule out malignancies.^13^, ^15^, ^19^ However, the studies mainly belong to pediatric populations from Europe, Australia, and America, preventing the generalization of the findings to different geographical regions.^17^

The aim of this study was to assess the clinical and dermoscopic features of acquired melanocytic nevi in Turkish children and adolescents and investigate the influence of demographic, constitutional, and environmental factors on these lesions.

## Methods

In this cross-sectional, single-center study, patients aged < 18 years admitted to the dermatology outpatient clinic of our tertiary referral center in the Western Turkey region were invited for melanocytic nevi examination with the consent of their legal guardians between January and June 2023. Patients with at least one lesion of any size compatible with melanocytic nevus both clinically and dermoscopically were consecutively enrolled in the study. Patients with a history of prolonged solar exposure four weeks prior to the examination, nevi of the nail unit, genital or mucosal region, congenital nevi diagnosed based on the morphology and presence during the first year of life, suspicious lesions necessitating biopsy to exclude malignancy, and special nevi such as Spitz nevus, agminated nevus, halo nevus or blue nevus were excluded.

The verbal and written consents were gathered from the legal guardians of the participants. Demographic findings (gender, age), personal and family history of skin cancer, sun protection practices by means of sunscreen use, and history of sunburn (in terms of number and severity [mild-moderate or severe characterized by the presence of erythema or painful blisters, respectively]) were recorded.

Participants were evaluated in two age groups: children (patients aged ≤ 10-years) and adolescents (patients aged > 10-years). They were examined regarding their eye and hair color. Skin phototype was assessed according to the Fitzpatrick scale.^26^

Melanocytic nevi were examined in terms of number, size (diameter), and anatomical region. The dermoscopic evaluation of melanocytic nevi was first performed with a hand-held dermatoscope (DermLite DL4®, 3Gen, California, USA), followed by the documentation with a digital camera (Canon EOS 5D®, Canon Inc., Tokyo, Japan) attached to a dermatoscopic lens (DermLite FOTO II Pro®, 3Gen, California, USA). Three dermatologists evaluated the lesions independently.

**Figure 1:**
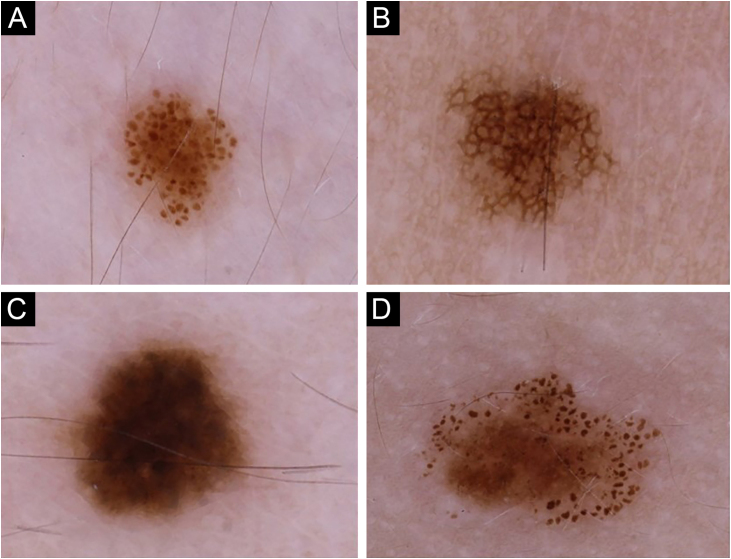
Nevi of the participants exhibiting four major dermoscopic patterns: (A) Globular, (B) Reticular, (C) Homogeneous, and (D) Complex.

Dermoscopic patterns were categorized into four major groups^27^: globular, reticular, homogeneous, and complex (Fig. 1), the latter being a combination of globular, reticular or homogeneous patterns,^28^ comprising globular-reticular, globular-homogeneous, and reticular-homogeneous. For each individual, the pattern observed in more than 40% of melanocytic nevi was accepted as the predominant dermoscopic pattern.^27^ The individuals not complying with this criterion were assigned to have an unspecified pattern. The nevi on the scalp and acral sites were only included in the total nevus count but evaluated separately in terms of dermoscopic features while determining the predominant dermoscopic pattern.

IBM SPSS® Statistics Version 26 was used to store and analyze the data. Shapiro-Wilk test was used to evaluate the normality of variable distribution. Descriptive statistics were calculated as mean ± standard deviation and median [minimum‒maximum] values for continuous variables and as frequency and percentage for categorical variables. The Chi-Square test or Fisher’s exact test was used to assess the differences in the distribution of categorical variables between two independent groups. The Mann-Whitney *U* test was used for the comparison of non-normally distributed groups. The p-value less than 0.05 was considered statistically significant.

The study has been approved by the institutional ethical committee (approval number: 2022/14-24) and conducted in accordance with the Declaration of Helsinki.

## Results

A total of 574 acquired melanocytic nevi of 100 participants were evaluated. The demographic and phenotypical characteristics of the study population are summarized in Table 1. The study group consisted of 50 females and 50 males with median ages of 9.5 and 11.5 years, respectively (p = 0.41). The median age and the distributions of gender and skin phototype were similar in both groups who used sunscreen and those who never used it (Table 2).

**Table 1:** Demographic, phenotypical and clinical characteristics of children and adolescents with acquired melanocytic nevi (n = 100).

Characteristics of participants	
Gender, female: male (ratio)	50:50 (1:1)
Age, years, median (range)	10 (2‒17)
Age group, n	
Children (≤ 10 years)	51
Adolescent (> 10 years)	49
History of skin cancer, n	0
Family history of skin cancer, n	3 (nonmelanoma skin cancer in all cases)
Eye colour, n	
Dark (Brown / black)	94
Light (Blue / green / hazel)	6
Hair colour, n	
Dark brown / black	79
Light brown / blonde	21
Skin phototype, n	
I	4
II	47
III	41
IV	8
Sunscreen use, n	
Never	40
Only during summer	42
Regularly	18
Previous history of sunburn, n	
Never	94
Mild-moderate	6
Severe	0
Number of melanocytic nevi, median (range)	4 (1‒38), (mean ± SD: 5.8 ± 5.1)
Predominant dermoscopic pattern of melanocytic nevi	Globular (n = 57), unspecified (n = 13), homogeneous (n = 12), complex (n = 10), reticular (n = 8)

SD, Standard Deviation.

**Table 2:** Comparison of participants using sunscreen and those who never used regarding gender, age and skin phototype.

	Using sunscreen (n = 60)	Not using sunscreen (n = 40)	p-value
**Gender, n (%)**			0.102^a^
Female	34 (56.7)	16 (40.0)	
Male	26 (43.3)	24 (60.0)	
**Age, years, median**	10	11	0.667^b^
**Skin phototype, n (%)**			0.165^a^
I‒II	34 (56.7)	17 (42.5)	
III‒IV	26 (43.3)	23 (57.5)	

a Chi-Square test.

b Mann-Whitney U test.

A significantly higher median number of melanocytic nevi was detected in adolescents compared to children (6 vs. 4; p < 0.05). On the other hand, the median nevi number did not show any difference regarding gender (female 4.5 vs. male 4; p = 0.424), skin phototype (type I‒II 5 vs. type III‒IV 4; p = 0.155) or sunscreen use (present 4 vs. not present 5; p = 0.345).

The upper extremities were the most common anatomical region harboring melanocytic nevi in 68 participants, followed by the trunk (n = 67) and face (n = 50) (Table 3). The prevalence of melanocytic nevi on the upper extremities was significantly higher in females than in males [80% (n = 40/50) vs. 56% (n = 28/50); p < 0.05]. The trunk was involved more frequently in males, albeit with no statistical significance (Table 4).

**Table 3:** Number, size and dermoscopic patterns (globular, reticular, homogeneous or complex^28^) of acquired melanocytic nevi according to anatomical distribution.

Anatomical region	Number of participants, n	Total nevus number in each anatomical region, n	Nevus number median (range)	Nevus size mm mean ± SD	Dermoscopic pattern of nevus			
					Globular, n (%)	Reticular, n (%)	Homogeneous n (%)	Complex, n (%)
Scalp	10	13	1 (1‒2)	3.4 ± 2.1	7 (53.8)	0 (0)	6 (46.2)	0 (0)
Face	50	121	2 (1‒10)	1.9 ± 0.9	81 (66.9)	0 (0)	40 (33.1)	0 (0)
Neck	17	45	2 (1‒7)	2.4 ± 1.3	41 (91.1)	0 (0)	4 (8.9)	0 (0)
Upper extremities	68	203	2 (1‒12)	2.3 ± 1.0	88 (43.3)	57 (28.1)	33 (16.3)	25 (12.3) [Globular-reticular (n = 15), globular-homogeneous (n = 9), reticular-homogeneous (n = 1)]
Trunk	67	154	2 (1‒15)	3.0 ± 1.8	104 (67.5)	21 (13.6)	9 (5.8)	20 (13.0) [Globular-homogeneous (n = 12), globular-reticular (n = 8)]
Lower extremities	17	32	1 (1‒6)	3.0 ± 2.1	9 (28.1)	9 (28.1)	12 (37.5)	2 (6.3) [Globular-reticular (n = 2)]
Acral	6	6	1	2.1 ± 1.6	**Dermoscopic pattern of nevus**			
					**Parallel furrow, n (%)**		**Fibrillar, n (%)**	**Lattice-like, n (%)**
					2 (33.3)		2 (33.3)	2 (33.3)

SD, Standard Deviation.

**Table 4:** Anatomical distribution of acquired melanocytic nevi with respect to gender (Statistically significant values are highlighted in bold).

	Female (n = 50)	Male (n = 50)	p-value
**Scalp, n (%)**	4 (8)	6 (12)	0.51^a^
**Face, n (%)**	26 (52)	24 (48)	0.69^a^
**Neck, n (%)**	7 (14)	10 (20)	0.42^a^
**Upper extremities, n (%)**	40 (80)	28 (56)	**0.01**^a^
**Trunk, n (%)**	29 (58)	38 (76)	0.06^a^
**Lower extremities, n (%)**	8 (16)	9 (18)	0.79^a^

a Chi-Square test.

The globular pattern was the most frequent predominant dermoscopic pattern (n = 57) (Fig. 1a) (Table 1). In 13 patients, no specific dermoscopic pattern predominated, while complex patterns were detected in the majority of nevi in 10 patients [globular-reticular (n = 7) and globular-homogeneous (n = 3)].

**Figure 2:**
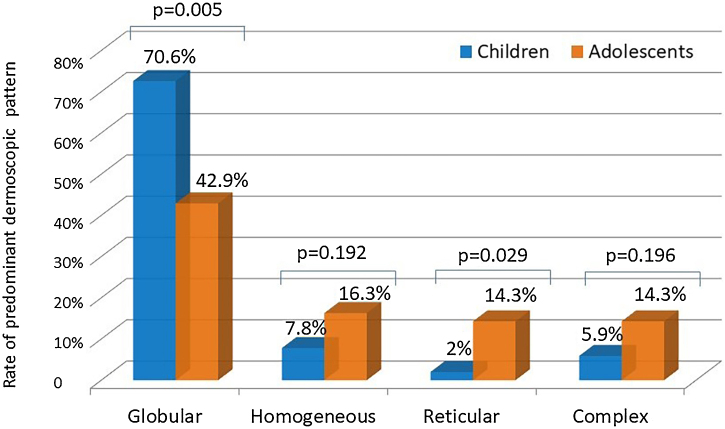
Comparison of the rates of predominant dermoscopic patterns in children and adolescents (Chi-Square test).

Even though both age groups had a globular pattern as the major predominant dermoscopic pattern, adolescents showed a significantly lower rate of this pattern than children (Fig. 2). On the other hand, a remarkable increase in the reticular pattern was noted in adolescents (Fig. 2). No difference in the predominant dermoscopic pattern was observed concerning gender, skin phototype, or sunscreen use (Table 5).

**Table 5:** Distribution of predominant dermoscopic pattern regarding gender, skin phototype and sunscreen use.

Predominant dermoscopic pattern	Gender			Skin phototype			Using sunscreen		
	Female	Male	p-value	I‒II	III‒IV	p-value	Yes	No	p-value
	(n = 50)	(n = 50)		(n = 51)	(n = 49)		(n = 60)	(n = 40)	
Globular, n (%)	28 (56.0)	29 (58.0)	0.840^a^	31 (60.8)	26 (53.1)	0.435^a^	38 (63.3)	19 (47.5)	0.117^a^
Reticular, n (%)	4 (8.0)	4 (8.0)	1.000^b^	4 (7.8)	4 (8.2)	1.000^b^	3 (5.0)	5 (12.5)	0.261^b^
Homogeneous, n (%)	7 (14.0)	5 (10.0)	0.538^a^	3 (5.9)	9 (18.4)	0.055^a^	5 (8.3)	7 (17.5)	0.213^b^
Complex, n (%)	6 (12.0)	4 (8.0)	0.505^a^	7 (13.7)	3 (6.1)	0.319^b^	5 (8.3)	5 (12.5)	0.515^b^
Unspecified, n (%)	5 (10.0)	8 (16.0)	0.372^a^	6 (11.8)	7 (14.3)	0.708^a^	9 (15.0)	4 (10.0)	0.466^a^

a Chi-Square test.

b Fisher’s exact test.

Globular pattern was the main dermoscopic pattern in any anatomical location except lower extremities where homogeneous pattern predominated (Table 3). Additionally, the globular and homogeneous patterns detected in participants with facial nevi were accompanied by a pseudo-network pattern in 86% (n = 43/50) of these individuals.

## Discussion

The evidence on the clinical and dermoscopic characteristics of melanocytic nevi in the young population is growing.^3^, ^4^, ^5^, ^6^, ^7^, ^8^, ^9^, ^10^, ^11^, ^12^, ^13^, ^14^, ^15^, ^16^, ^17^, ^18^, ^19^, ^20^, ^21^, ^22^, ^23^, ^24^, ^25^ However, it is still challenging to compare these studies due to differences in age groups and skin phototypes of participants, the frequency and degree of solar exposure depending on the geographical region where the study was conducted. Furthermore, some studies only included nevi from specific anatomical sites, such as the back,^21^, ^23^ as some examined lesions larger than 2 mm in diameter only.^9^, ^22^

### Geographical region

In studies evaluating total body nevus count in children and adolescents, the mean nevus number showed a wide range from one to seventy-odd,^6^, ^11^ partly explained by the latitude. A comparative study conducted on high school children of European ancestry living at different latitudes (from Australia and Scotland) showed a significantly higher number of nevi in children from Australia.^5^ A similar difference was noted in a study investigating nevi development during the first three years of life in two cohorts of children from Australia and Scotland.^29^ English et al. used erythemal irradiance as a measure of ambient ultraviolet exposure instead of latitude in their study and demonstrated that 6-year-old children who had been living in Perth, Australia since their birth had a lower nevus count on the back compared to those moving to Perth from other regions with higher erythemal irradiance.^30^ A previous study on Turkish children and adolescents from Eastern Turkey reported a mean nevus count of 1.1,^11^ which was nearly six times lower than the value detected in our series. This difference among the regions of the same country could be linked to other possible contributing factors, such as the proximity to the sea coast, as our study population mainly resides along the seaside.

### Genetic factors

The total nevus count was also shown to be determined by genetic factors.^31^, ^32^ In addition, the association between specific genetic polymorphisms and particular dermoscopic patterns has recently been demonstrated in children.^33^

### Age

The increase in nevus count from childhood to adolescence is a well-established finding,^3^, ^4^, ^12^, ^13^^,^^17^, ^19^ also supported by our series. The globular pattern was reported as the major predominant dermoscopic pattern in children.^3^, ^19^ Zalaudek et al. indicated the globular pattern as the main pattern observed in participants aged 3‒20 years in their study evaluating the dermoscopic patterns of trunkal nevi. The authors also observed a significant rise in reticular pattern prevalence in adolescence.^19^ Despite being the most common pattern in both age groups, the globular pattern was more prevalent in children than in adolescents in our series due to the increasing trend of reticular pattern with advancing age. According to the dual concept of nevogenesis, the nevi with a globular pattern represent genetically determined delayed-onset congenital nevi, mainly appearing during early childhood and persisting lifelong. Nevi with a reticular pattern, on the other hand, are indicated as “true” acquired melanocytic lesions that emerge after puberty in response to ultraviolet radiation and disappear eventually.^15^, ^34^ This theory was also supported by recent longitudinal studies, where a switch from a globular to a reticular pattern was shown to be infrequent.^18^, ^25^ On the other hand, Italian researchers reported a remarkable rate of transition from one pattern to another (mainly from globular to globular-reticular), particularly in the 3‒6 years age group.^20^

### Gender

Some authors indicated no gender-related difference in total nevus count,^3^, ^14^ similar to our findings. In contrast, males were generally reported to have a higher number of nevi.^9^, ^17^, ^22^ A recent report noted this difference to be pronounced following puberty.^12^

### Skin phototype

Individuals with fair skin phototypes were demonstrated to possess nevi with a predominant globular pattern and have higher numbers of nevi than those with darker skin phototypes, mainly associated with a reticular pattern.^3^, ^9^, ^10^, ^12^^,^^16^, ^21^ Thus, in studies conducted in pediatric populations with darker skin phototypes, the reticular pattern prevailed.^21^, ^24^ This was attributed to high melanocytic activity and increased melanin transfer to keratinocytes, resulting in an augmented epidermal rete ridge pattern seen as a reticular pattern in these individuals.^16^ The lack of any significant impact of skin phototype on nevus count or predominant dermoscopic pattern in our series might be linked to the unbalanced distribution of participants with different skin phototypes, most classified as phototype II or III.

### Anatomical site

Both genetic factors and ultraviolet radiation influence the anatomical distribution of nevi.^35^ Intermittent ultraviolet exposure was associated with a higher nevus count compared to chronic ultraviolet exposure.^4^, ^9^, ^35^ This was related to the nevogenic effect of intermittent exposure on melanocytes as well as the protective effect of chronic exposure against nevi development.^8^, ^9^, ^35^ Compatible with these findings, the body sites exposed to ultraviolet radiation intermittently (upper extremities and trunk) were the most frequently involved regions in our study group.

In several studies, a significantly increased number of melanocytic nevi were reported on the trunk in males and on the extremities in females,^3^, ^4^ in accordance with our findings. This distribution pattern is similar to that observed for nevi and melanoma in adults,^4^, ^35^ although the direct relationship between nevus count and melanoma development in a specific anatomical site is unclear.^36^ Differences in sun exposure patterns due to clothing style and outdoor activities were held responsible for this gender-related difference^3^ as well as hormonal effects.^9^ However, a similar nevi distribution pattern was observed in Canadian Hutterite children with limited ultraviolet exposure due to their religious clothing.^7^ The authors of this study, who identified this pattern even in prepubertal children, suggested a possible regional biological difference in melanocytes rather than ultraviolet radiation or hormonal effect.^7^ Accordingly, the high nevus count on lower extremities in females has recently been shown to be influenced by gender-specific genetic effects.^37^

Melanocytic nevi showed distinct dermoscopic patterns depending on the anatomical site.^22^, ^23^ In a cross-sectional analysis of the back and leg nevi of adolescents, conducted as a part of a population-based study, the globular pattern was observed more frequently on the back than the reticular pattern, which was more prevalent on the legs.^23^ Similarly, in a series from Italy, lesions from the neck, trunk and upper extremities showed a higher rate of globular pattern, whereas the reticular pattern was more prevalent on the lower extremities.^22^ This was attributed to the cephalad-to-caudal migration and arrest of melanoblasts in the dermis during embryogenesis, which resulted in the formation of globular nevi. The reticular nevi, on the other hand, are thought to derive from epidermal melanocytes following intermittent ultraviolet radiation on sun-exposed anatomical sites.^23^ In line with these findings, the globular pattern predominated on the trunk and upper parts of the body in our series. However, the homogeneous pattern was the most common pattern recorded in the lower extremities instead of the reticular. This might be explained by the inclusion of all nevi of any size in contrast to the studies above in which up to six nevi per participant^23^ or nevi larger than 2 mm in diameter were selected from each anatomical region.^22^

### Ultraviolet exposure

Ultraviolet exposure^8^, ^10^ and a history of sunburn^3^, ^8^, ^12^, ^17^ were associated with an increased number of melanocytic nevi. The evidence regarding the impact of sunscreen use on the total nevus count during childhood is controversial.^12^, ^38^ However, studies varied in methodology, including study design, age groups, and the lowest limit of the examined nevus size. Furthermore, the sun protection factor, water resistance, and details about sunscreen application were not always questioned.^12^, ^38^ The reports indicating increased nevus counts with sunscreen use could be explained by the use of sunscreen mainly in children with fair skin phototypes who are more prone to sunburn and already have an increased propensity for nevi development. The prolonged sun exposure of children following sunscreen application and the parents’ biased responses regarding their actual sunscreen use strategy for their children due to social pressure might be other causes.^8^, ^12^, ^38^

A higher number of globular and lower number of reticular nevi were associated with the use of sunscreen with SPF > 30 in a recent study from Greece.^3^ This observation further supports the relation between reticular pattern and ultraviolet exposure proposed in the dual concept of nevogenesis. In our present study, no impact of sunscreen use on the number or dermoscopic pattern of melanocytic nevi was observed, which might also be related to the misleading factors discussed above.

The small sample size was the main limitation of our study. Moreover, there was a possible response bias due to parents’ answers regarding the use of sunscreen and the history of sunburn. The limited number of parents reporting a sunburn history in their children hindered evaluating its impact on nevus number and dermoscopic pattern. Our study’s main strength was the complete body examination of participants and the inclusion of all nevi regardless of size. Furthermore, the study group represents the general Turkish pediatric population as the participants were patients from a general outpatient clinic, not a follow-up clinic for melanocytic lesions.

## Conclusions

In conclusion, nevogenesis is a complex process due to the significant interplay between genetic and environmental factors. The age and anatomical site were the most relevant factors influencing the number and dermoscopic patterns of nevi in our study. The predominance of globular pattern in childhood and the increasing rate of reticular pattern in adolescence support the dual concept of nevogenesis.^34^ The dermoscopic patterns differing in terms of anatomical sites also confirm the presence of distinct developmental pathways. The gender-related distribution pattern of nevi, without any effect of sunscreen use on either nevus count or dermoscopic pattern, further suggests a genetic predisposition.

## Financial support

None declared.

## Authors’ contributions

Zeynep Keskinkaya: The study concept and design, data collection, analysis and interpretation, statistical analysis, writing of the manuscript, critical review of important intellectual content, effective participation in the research guidance, intellectual participation in the propaedeutic and/or therapeutic conduct of the studied cases, critical review of the literature, final approval of the final version of the manuscript.

Özge Kaya: The study concept and design, analysis and interpretation of data, writing of the manuscript, critical review of important intellectual content, intellectual participation in the propaedeutic and/or therapeutic conduct of the studied cases, final approval of the final version of the manuscript.

Selda Işık Mermutlu: The study concept and design, analysis and interpretation of data, writing of the manuscript, critical review of important intellectual content, intellectual participation in the propaedeutic and/or therapeutic conduct of the studied cases, final approval of the final version of the manuscript.

Hilay Garipcan Karaemir: The study concept and design, data collection, analysis and interpretation, writing of the manuscript, critical review of important intellectual content, intellectual participation in the propaedeutic and/or therapeutic conduct of the studied cases, final approval of the final version of the manuscript.

Sevilay Oğuz Kılıç: The study concept and design, analysis and interpretation of data, writing of the manuscript, critical review of important intellectual content, intellectual participation in the propaedeutic and/or therapeutic conduct of the studied cases, final approval of the final version of the manuscript.

## Conflicts of interest

None declared.
